# Anticonvulsant effect of pterostilbene and its influence on the anxiety- and depression-like behavior in the pentetrazol-kindled mice: behavioral, biochemical, and molecular studies

**DOI:** 10.1007/s00213-021-05933-5

**Published:** 2021-08-01

**Authors:** Dorota Nieoczym, Katarzyna Socała, Agnieszka Zelek-Molik, Mateusz Pieróg, Katarzyna Przejczowska-Pomierny, Małgorzata Szafarz, Elżbieta Wyska, Irena Nalepa, Piotr Wlaź

**Affiliations:** 1 Department of Animal Physiology and Pharmacology, Institute of Biological Sciences, Maria Curie-Skłodowska University, Akademicka 19, 20-033 Lublin, Poland; 2 Department of Brain Biochemistry, Maj Institute of Pharmacology, Polish Academy of Sciences, Smętna 12, 31-343 Kraków, Poland; 3 Department of Pharmacokinetics and Physical Pharmacy, Faculty of Pharmacy, Jagiellonian University Medical College, Medyczna 9, 30-688 Kraków, Poland

**Keywords:** Pterostilbene, PTZ-induced kindling, Elevated plus maze test, Forced swim test, Epilepsy, Mice

## Abstract

**Rationale:**

Pterostilbene is the 3,5-dimethoxy derivative of resveratrol with numerous beneficial effects including neuroprotective properties. Experimental studies revealed its anticonvulsant action in the acute seizure tests.

**Objectives:**

The purpose of the present study was to evaluate the effect of pterostilbene in the pentetrazol (PTZ)–induced kindling model of epilepsy in mice as well as to assess some possible mechanisms of its anticonvulsant action in this model.

**Methods:**

Mice were repeatedly treated with pterostilbene (50–200 mg/kg) and its effect on the development of seizure activity in the PTZ kindling was estimated. Influence of pterostilbene on the locomotor activity and anxiety- and depression-like behavior in the PTZ-kindled mice was also assessed. To understand the possible mechanisms of anticonvulsant activity of pterostilbene, γ-aminobutyric acid (GABA) and glutamate concentrations in the prefrontal cortex and hippocampus of the PTZ-kindled mice were measured using LC–MS/MS method. Moreover, mRNA expression of BDNF, TNF-α, IL-1β, IL-6, *GABRA1A*, and *GRIN2B* was determined by RT-qPCR technique.

**Results:**

We found that pterostilbene at a dose of 200 mg/kg considerably reduced seizure activity but did not influence the locomotor activity and depression- and anxiety-like behavior in the PTZ-kindled mice. In the prefrontal cortex and hippocampus, pterostilbene reversed the kindling-induced decrease of GABA concentration. Neither in the prefrontal cortex nor hippocampus pterostilbene affected mRNA expression of IL-1β, IL-6, *GABRA1A*, and *GRIN2B* augmented by PTZ kindling. Pterostilbene at a dose of 100 mg/kg significantly decreased BDNF and TNF-α mRNA expression in the hippocampus of the PTZ-kindled mice.

**Conclusions:**

Although further studies are necessary to understand the mechanism of anticonvulsant properties of pterostilbene, our findings suggest that it might be considered a candidate for a new antiseizure drug.

## Introduction

Epilepsy is a chronic neurological disorder affecting 65 million people in the world (Moshe et al. 2015). It is characterized by unprovoked and recurrent seizures resulted from the disruption of the electrical activity of neurons (Fisher et al. 2014). In the clinic, seizures might manifest as only short periods of altered consciousness, abnormal sensory, autonomic or cognitive functions, or convulsive seizures with clonic and/or tonic components. The appearance of seizures depends on the brain structures and size of the area of the brain where abnormal discharges occur (Avoli et al. 2002; D'Antuono et al. 2002; Tancredi et al. 2000). The imbalance between the excitatory and inhibitory neurotransmission in the central nervous system is considered the main reason of these abnormalities and components of these systems are the main targets for antiseizure/antiepileptic therapy (Lasoń et al. 2013).

Despite the wide availability of antiseizure drugs, the effective treatment of epileptic disorders remains problematic due to several reasons. Firstly, drug-resistant epilepsy (defined by the International League Against Epilepsy as the lack of sustained seizure freedom after using two well-tolerated and appropriately chosen antiseizure drug schedules) is diagnosed in about 30% of epileptic patients (Billakota et al. 2020). The second important problem in epilepsy treatment is frequent and severe adverse effects of antiseizure medication which are the reason for the discontinuation of pharmacotherapy in about 25% of patients (Perucca and Gilliam 2012). Thirdly, current antiseizure drugs are used mainly to suppress already diagnosed seizures but they do not prevent the development of seizures in patients who are at risk of epilepsy (Billakota et al. 2020). Moreover, epileptic disorders are often associated with some psychiatric comorbidities such as depression and/or anxiety symptoms (Scott et al. 2017; Thapar et al. 2009). In the view of the above, there is a need to identify new medications that enable not only successful treatment of patients with epileptic disorders and those who are endangered by the disease development, but also those with comorbid anxiety and depression.

Pterostilbene (3,5-dimethoxy-4′-hydroxystilbene) is a naturally derived non-flavonoid polyphenol structurally similar to resveratrol. This compound has gained much interest in the last decade due to its numerous beneficial pharmacological properties including antioxidant, anti-inflammatory, antidiabetic, chemopreventive, and chemotherapeutic effects (Lin et al. 2020). Numerous studies revealed that pterostilbene crosses the blood–brain barrier and affects central nervous system function (Poulose et al. 2015). It improved cognition and neuronal functioning during ageing (Chang et al. 2012; La Spina et al. 2019) as well as showed anxiolytic- (Al Rahim et al. 2013) and antidepressant-like (Yang et al. 2019) activity in animal models. Our previous studies revealed that pterostilbene has anticonvulsant effects in acute seizure tests both in mice and zebrafish larvae (Nieoczym et al. 2019a) as well as enhances anticonvulsant activity of some antiseizure drugs in mice (Nieoczym et al. 2019b). The present study aimed to evaluate the activity of pterostilbene in the pentetrazol (PTZ)–induced kindling in mice which is a reliable and widely used model of epilepsy (Alachkar et al. 2020b; Kamiński et al. 2020; Socała et al. 2019). We also investigated the influence of pterostilbene on the anxiety- and depression-like behavior in the PTZ-kindled mice. To evaluate possible mechanisms of anticonvulsant action of pterostilbene, GABA and glutamate concentrations were determined in the prefrontal cortex and hippocampus of mice. These brain structures were selected for the analysis due to their verified participation in the PTZ-induced kindling (Szyndler et al. 2009). Reverse transcription and the quantitative polymerase chain reaction (RT-qPCR) were used to measure the expression of mRNA of brain-derived neurotrophic factor (BDNF), pro-inflammatory cytokines, i.e., tumor necrosis factor-α (TNF-α), interleukin (IL)-1β, and IL-6, as well as GABA_A_ and NMDA receptors subunits (i.e., *GABRA1A* and *GRIN2B* genes, respectively).

## Material and methods

### Animals

The study was carried out on 95 adult male Swiss mice, weighing 23–28 g. Animals were obtained from the Centre for Experimental Medicine at the Medical University of Lublin. The animals were housed in the standard polycarbonate cages (7–8 mice/cage) under strictly controlled conditions including ambient temperature 21–23 °C, relative humidity 45–65%, and 12/12 light/dark cycle with the light on at 6:00 a.m., with free access to chow pellets and tap water. Each experimental group consisted of 10–15 animals. The mice were used after at least 1 week of acclimatization. All behavioral experiments were performed at the same time of day (between 8:00 a.m. and 3:00 p.m.) to minimize circadian influences. Control and drug experiments were always done on the same day to avoid day-to-day variations in convulsive susceptibility. All procedures were conducted in accordance with the European Union Directive of 22 September 2010 (2010/63/EU) and Polish legislation acts concerning animal experimentations. The experimental procedures and protocols were approved by the Local Ethics Committee in Lublin (1/2019).

### Drugs

The following compounds were used in the study: pterostilbene (Toronto Research Chemicals Inc., Toronto, ON, Canada), PTZ (Sigma-Aldrich, St. Louis, MO, USA), and valproic acid (VPA, as sodium salt; Sigma-Aldrich Co., St. Louis, MO, USA). Pterostilbene was suspended in a 5% solution of Tween 80 (POCH, Gliwice, Poland) in normal saline, while VPA was dissolved in saline. Pterostilbene was administered at doses of 50, 100, and 200 mg/kg and VPA at a dose of 150 mg/kg. Both compounds were injected intraperitoneally (*ip*), 30 min before each PTZ treatment. The time of drug administration was selected based on the results of the previous studies (Nieoczym et al. 2019a, b). The negative control group was treated with a 5% solution of Tween 80, 30 min before the PTZ injection. VPA—a standard anticonvulsant medication—was used as a positive control. All solutions and suspensions were administered at a volume of 10 mL/kg of body weight.

### PTZ kindling procedure

PTZ-induced kindling was established and described in our previous studies (Kamiński et al. 2020; Socała et al. 2019). To induce kindling, mice were treated *ip* with PTZ at a subconvulsive dose of 35 mg/kg. PTZ administration was repeated three times a week with intervals of at least 48 h. Mice were divided into 6 experimental groups, i.e., (1) normal control – 5% Tween 80 + saline-treated; (2) PTZ control – 5% Tween 80 + PTZ-treated; (3) positive control – VPA 150 mg/kg + PTZ-treated; (4) pterostilbene 50 mg/kg + PTZ-treated; (5) pterostilbene 100 mg/kg + PTZ-treated; (6) pterostilbene 200 mg/kg + PTZ-treated. The PTZ control, positive control, and pterostilbene-treated groups consisted of 15 mice, while the normal control group contained 20 animals. PTZ solution was administered 30 min after 5% Tween, VPA, or pterostilbene injection. Animals received 21 injections of PTZ, while the normal control group (non-kindled) obtained 21 applications of saline. Following PTZ treatment, animals were placed in the transparent box and observed for 30 min to assess the seizure score using the modified Racine’s scale (Racine 1972) that defines the following: stage 0, normal behavior; stage 1, immobilization, vibrissae twitching, ear and eye twitches; stage 2, myoclonic jerks; stage 3, unilateral forelimb clonus; stage 4, rearing with bilateral forelimb clonus; stage 5, generalized tonic–clonic seizures with loss of posture control; stage 6, fore- and hindlimb tonic extension. The mean seizure severity score was calculated in the experimental group after each PTZ injection. Animals with three consecutive 5 stages were considered to be fully kindled.

Twenty-four hours after the last PTZ injection, locomotor activity, anxiety- and depression-like behavior were assessed in PTZ-kindled mice and, after that, animals were decapitated to collect brain structures, i.e., the prefrontal cortex and hippocampus, for biochemical and molecular analyses.

**Fig. 1:**
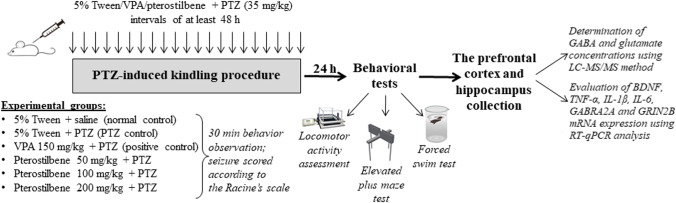
Schematic presentation of the experimental design. Six experimental groups were established in the study, i.e., (1) normal control – 5% Tween 80 + saline-treated; (2) PTZ control – 5% Tween 80 + PTZ-treated; (3) positive control – VPA 150 mg/kg + PTZ-treated; (4) pterostilbene 50 mg/kg + PTZ-treated; (5) pterostilbene 100 mg/kg + PTZ-treated; (6) pterostilbene 200 mg/kg + PTZ-treated. To induce kindling, mice were treated *ip* with PTZ at a dose of 35 mg/kg three times a week with intervals of at least 48 h (total of 21 injections). Pterostilbene, VPA, and 5% Tween were administered 30 min before each PTZ injection. Following PTZ treatment, mice were placed individually in the transparent box and observed for 30 min to estimate seizure score according to the Racine’s scale. Twenty-four hours after the last PTZ administration, behavioral tests were conducted and, after that, the prefrontal cortex and hippocampus were collected for LC–MS/MS and RT-qPCR analysis. PTZ, pentetrazol; VPA, valproic acid

The experimental design is presented in Fig. 1.

### Assessment of the locomotor activity

The automated infrared beam-based system was used to monitor the spontaneous locomotor activity of mice (IR Actimeter, Panlab/Harvard Apparatus, Barcelona, Spain). The apparatus consists of a black square arena (25 cm × 25 cm) surrounded by transparent walls (height 35 cm) and frame equipped with infrared beams (16 × 16). Breaks of the photo beams were recorded by a computer system (SedaCom32 computer software) and the interruption counts were used as a measure of horizontal locomotor activity of mice. Spontaneous locomotor activity was measured for 5 min.

### Assessment of the anxiety-like behavior in the elevated plus maze test

Anxiety-like behavior was measured using the method described by Lister (1987). The apparatus for the plus maze test was made of a matte black Plexiglas and consisted of two open (30 cm × 5 cm) and two closed arms (30 cm × 5 cm × 15 cm) connected by the open central squire platform (5 cm × 5 cm). The plus maze was located at the height of 38 cm above the floor and illuminated by the dim red light. During the experiment, each mouse was placed on the central platform facing one of the open arms and allows to freely explore the maze for 5 min. The behavior and movement of animals during the test were video recorded and ANY-maze software (version 4.82, Stoelting Co., Chicago, IL, USA) was used to register the total number of entries and the total time spent in both kinds of arms. Results of the test were presented as the percentage of the entries into the open arms and the percentage of the time spent in these arms. Between trials, the apparatus was cleaned with a 0.1% solution of acetic acid to remove odor marks.

### Estimation of the depressive-like behavior in the forced swim test

The forced swim test was carried out according to the method described by Porsolt et al. (1977). Mice were placed individually in a glass cylinder (25 cm high; 10 cm diameter) filled to a level of 11 cm with tap water. The water temperature was kept at 24 ± 1 °C. Mice were placed into the center of the cylinder for 6 min. The immobility of mice was recorded during the last 4 min of the test using cumulative stopwatches. The mouse was defined immobile when it floated in the water without struggling and making only necessary movements to keep its head above the water and no attempts to escape. After each trial, the water in the cylinder was replaced with fresh water. Data obtained in the forced swim test were expressed as the mean (in s) ± standard error of the mean (SEM) immobility time in each experimental group.

### Determination of GABA and glutamate concentrations in the prefrontal cortex and hippocampus

Concentrations of GABA and glutamate in mice prefrontal cortex and hippocampus were measured by a liquid chromatography tandem mass spectrometry (LC–MS/MS) method. Standards of both analytes were purchased from Toronto Research Chemicals Inc. (Toronto, ON, Canada). The stock standard solutions of GABA and glutamate were prepared in methanol and deionized water, respectively, and stored at 4 °C. A series of solution mixtures of desired concentrations were prepared by suitable dilutions of the stock solutions. Before analysis, murine brains were homogenized in distilled water at the ratio of 50 µL/mg using a hand-held pestle and glass tube homogenizer (Potter–Elvehjem PTFE pestle and glass tube, Sigma-Aldrich). Homogenates were centrifuged at 8000 × *g* for 10 min at 4 °C and the supernatant was diluted 10 times with 0.1% formic acid in acetonitrile. After addition of isotope-labelled GABA–d_6_ and glutamate–d_5_ (Toronto Research Chemicals Inc., Toronto, ON, Canada) as internal standards (10 μL at the concentration of 500 ng/mL), samples (10 μL) were deproteinized with 80 μL of 0.1% formic acid in acetonitrile by shaking for 10 min (IKA Vibrax VXR, Germany) and then centrifuged for 5 min at the speed of 8000 × *g* (Eppendorf miniSpin centrifuge). The obtained supernatants were transferred into the autosampler vials. Chromatographic separation was carried out on XBridge™ HILIC analytical column (2.1 × 150 mm, 3.5 µm, Waters, Ireland) with the oven temperature set at 25 °C using the Excion LC AC HPLC system. The autosampler temperature was maintained at 15 °C and a sample volume of 2 μL was injected into the LC–MS/MS system. The mobile phase containing 0.1% formic acid in acetonitrile and 0.1% formic acid in water was mixed at a ratio of 70:30 and run at 0.3 mL/min. Mass spectrometric detection was performed on an Sciex QTRAP 4500 triple quadrupole mass spectrometer. Electrospray ionization (ESI) in the positive ion mode was used for ion production. The tandem mass spectrometer was operated at unit resolution in the selected reaction monitoring mode (SRM), monitoring the transition of the protonated molecular ions *m/z* 104 to 87 and *m/z* 104 to 69 for GABA and *m/z* 148 to 84 and *m/z* 148 to 102 for glutamate (first pair was used as an quantifier and the second for the identity verification—qualifier). For isotope labelled GABA–d_6_ and glutamate–d_5_ monitored pairs were *m/z* 110 to 93 and *m/z* 153 to 88, respectively. The mass spectrometric conditions were optimized for GABA and glutamate by continuous infusion of the standard solution at the rate of 7 μL/min using a Harvard infusion pump. The ion source temperature was maintained at 450 °C. The ionspray voltage was set at 5000 V. The curtain gas (CUR) was set at 40 psi and the collision gas (CAD) at Medium. Data acquisition and processing were accomplished using the Applied Biosystems Analyst version 1.7 software. The calibration curves were constructed by plotting the ratio of the peak area of the studied compound to internal standard versus drug concentration and generated by weighted (1/x‧x) linear regression analysis. Due to the high endogenous concentrations of GABA and glutamate and availability of the stable isotope standards, calibrations curves were constructed based on serial dilutions of the calibrators in water. The validated quantitation ranges for this method were within the expected concentration ranges, namely from 100 to 5000 µg/g of brain tissue with accuracy from 90.89 to 108.43% and from 90.48 to 111.36% for GABA and glutamate, respectively. No significant matrix effect was observed and there were no stability-related problems during the routine analysis of the samples. Protein concentrations were measured in all samples using the Bradford Protein Assay (Bio-Rad, Hercules, CA, USA) and the final results were presented as µg per mg of protein.

### Analysis of mRNA expression

Total RNA was isolated and purified and mRNA expression was assessed based on the previously described protocol (Zelek-Molik et al. 2019). The frozen tissue was placed in lysis buffer containing guanidinium thiocyanate (Zymo Research, USA) in a volume of 0.4 mL/20 mg of tissue and homogenized by high-speed shaking (30/s) in plastic tubes with stainless steel beads in a TissueLyser II apparatus (Qiagene, USA). Total RNA was purified by Quick-RNA MiniPrep (Zymo Research, USA) according to the manufacturer’s protocol. The quantity of RNA was determined spectrophotometrically at 260 nm and 260/280 nm (ND/1000 UV/Vis; Thermo FisherNanoDrop, USA), and its quality was confirmed by agarose gel electrophoresis. Reverse transcription and the quantitative polymerase chain reaction (RT-qPCR) were performed on the QuantStudio™12 K Flex system (Life Technologies, USA). The RT reaction was performed at a final volume of 20 μL with 300 ng of RNA (as a cDNA template) using the High Capacity cDNA Reverse Transcription Kit (Applied Biosystems, USA) according to the manufacturer’s protocol. Products of the RT reaction were amplified with the TaqMan Gene Expression Master Mix (Applied Biosystems, USA) in a total volume of 10 µl containing 1 × TaqMan Gene Expression Master Mix, 30 ng of cDNA (used as the PCR template), and 250 nM TaqMan probe labeled with FAM as the fluorescent dye. In these experiments, the following TaqMan probes (ThermoFisher Scientific, USA) were used as primers for specific genes: BDNF # Mm04230607_s1; TNF-α # Mm00443258_m1; IL-1β # Mm00434228_m1; IL-6 # Mm00446190_m1; Gabra1 # Mm00439046_m1; Grin2b # Mm00433820_m1; Hprt1 # Mm01324427_m1; b2M # Mm00437762_m1. The following standard qPCR protocol was used: 2 min at 50 °C, 10 min at 95 °C, and then 40 cycles for 15 s at 95 °C and 1 min at 60 °C. The threshold cycle value (Ct) for each sample was set in the exponential phase of PCR, and the ΔΔ Ct method was used to analyze the data. HPRT and/or B2m was used as a reference gene, and its expression was considered to be at a constant level in all experimental groups of animals.

### Statistical analysis

Seizure scores in the PTZ-induced kindling test were analyzed using two-way analysis of variance (ANOVA) with repeated measures followed by Dunnett’s multiple comparison test. One-way ANOVA with Dunnett’s post hoc test was employed to compare data from behavioral tests conducted after the PTZ kindling procedure, i.e., the forced swim test, the elevated plus maze test, and locomotor activity measurement, as well as results from the RT-PCR analysis. In Dunnett’s test, the control PTZ-kindled group was established as a control group because our intention was to check pterostilbene-induced changes in the kindled mice. GABA and glutamate concentrations in the prefrontal cortex and hippocampus were evaluated using one-way ANOVA with Tukey’s post hoc test. Differences were considered statistically significant if *p* ≤ 0.05.

The statistical tests were performed using GraphPad Prism (version 8) for Windows (GraphPad Software, San Diego, CA).

## Results

### Effect of repeated treatment with pterostilbene on the seizure severity in the PTZ kindling in mice

**Fig. 2:**
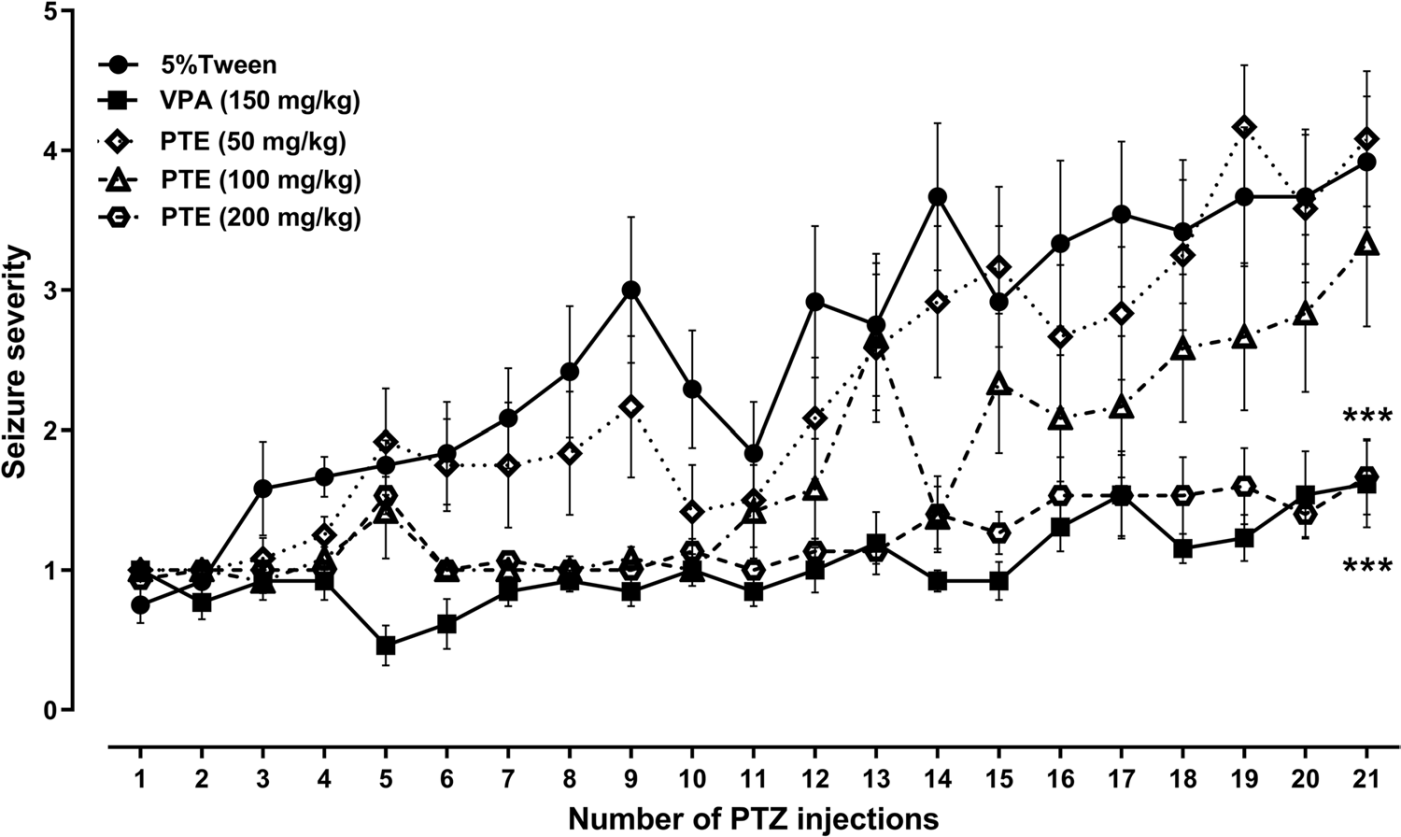
Effect of pterostilbene on the PTZ-induced kindling development in mice. PTE, pterostilbene; PTZ, pentetrazol; VPA, valproic acid. Data are presented as means ± SEM, *n* = 12–15 mice per group. Statistical analysis was performed using two-way ANOVA with repeated measures (number of PTZ injection, F(20,1239) = 23.00, *p* < 0.0001; treatment, F(4,1239) = 91.52, *p* < 0.0001; number of PTZ injections × treatment, F(80, 1239) = 2.013, *p* < 0.0001) followed by Dunnett’s multiple comparison test. ***p < 0.001 vs. control PTZ-kindled group

The PTZ kindling model in mice was established by repetitive *ip* administration of PTZ at a dose of 35 mg/kg on alternate days (21 injections). The consecutive injection of PTZ caused a progressive increase in the seizure score assessed according to the Racine’s scale. After the initial PTZ injections, seizure activity manifested as immobility and hardly visible myoclonic twitches classified as 1 and 2 seizure scores and afterwards turned into generalized tonic–clonic convulsions. The maximal medium seizure score of 3.92 ± 0.5 was reached in the control PTZ-kindled group after 21st PTZ injection. The effect of repeated treatment with pterostilbene at doses ranging from 50 to 200 mg/kg on the kindling development in mice is presented in Fig. 2 (two-way ANOVA with repeated measures: number of PTZ injections, F(20,1239) = 23.00, *p* < 0.0001; treatment, F(4,1239) = 91.52, *p* < 0.0001; number of PTZ injections × treatment, F(80,1239) = 2.013, *p* < 0.0001). Repeated treatment with pterostilbene at doses of 50 and 100 mg/kg did not significantly influence the mean seizure score noted after the last PTZ injection (*p* > 0.05 vs. control PTZ-kindled group). Statistically significant decrease in the seizure severity after 21st PTZ injection in comparison to the control PTZ-kindled group was noted both in the positive control (VPA-treated) group (*p* < 0.0001) as well as in the group of animals repeatedly treated with pterostilbene at a dose of 200 mg/kg (*p* < 0.0001). The final mean seizure score in the positive control group was 1.62 ± 0.3, while in the group of animals treated with pterostilbene at a dose of 200 mg/kg it was 1.67 ± 0.27.

### Effect of repeated treatment with pterostilbene on the locomotor activity in the PTZ-kindled mice

**Fig. 3:**
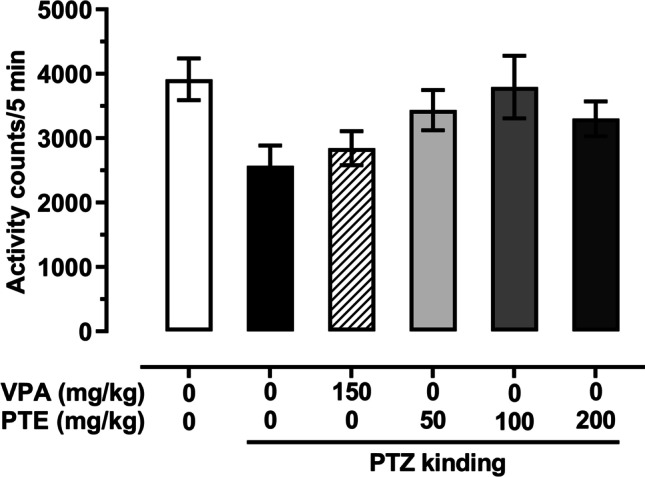
Effect of repeated treatment with pterostilbene on the locomotor activity in the PTZ-kindled mice. PTE, pterostilbene; PTZ, pentetrazol; VPA, valproic acid. Data are presented as means ± SEM, *n* = 9–15 mice per group. Statistical analysis was performed using one-way ANOVA (F(5,64) = 2.243, *p* < 0.061)

Statistical analysis of the obtained data revealed that the PTZ-induced kindling procedure did not change significantly spontaneous locomotor activity of mice (as compared to the saline-treated non-kindled control group). Moreover, repeated administration of VPA (150 mg/kg) or pterostilbene (50–200 mg/kg) did not significantly affect locomotor activity in mice submitted to the PTZ-induced kindling procedure (Fig. 3; one-way ANOVA: F(5,64) = 2.243, *p* < 0.061).

### Effect of repeated treatment with pterostilbene on the anxiety-like behavior in the PTZ-kindled mice

**Fig. 4:**
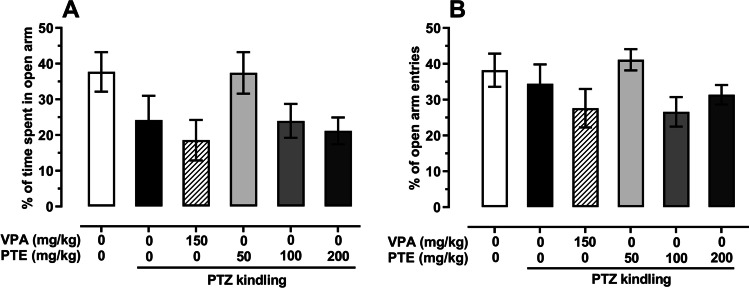
Effect of repeated treatment with pterostilbene on the percentage of time spent in the open arm (panel **A**) and on the percentage of open arm entries (panel **B**) in the elevated plus maze test of the PTZ-kindled mice. PTE, pterostilbene; PTZ, pentetrazol; VPA, valproic acid. Data are presented as means ± SEM, *n* = 9–15 mice per group. Statistical analysis was performed using one-way ANOVA (percentage of time spent in the open arm, F(5,64) = 2.235, *p* = 0.061; percentage of open arm entries, F(5,64) = 1.806, *p* = 0.124)

The mean percentage of time spent in the open arm of the elevated plus maze in the saline-treated non-kindled group was 37.69 ± 5.5, while in the control PTZ-kindled group it was reduced to 24.19 ± 6.8; however, this change was not statistically significant (*p* > 0.05). Moreover, repeated treatment with VPA (150 mg/kg) or pterostilbene (50–200 mg/kg) did not significantly influence the percentage of time spent in the open arm in the elevated plus maze test (*p* < 0.05; both in comparison to the control PTZ-kindled group and to the saline-treated non-kindled group). PTZ-induced kindling procedure also did not significantly affect the percentage of open arm entries of mice (*p* > 0.05). Statistical analysis did not reveal any significant influence of repeated injections of VPA (150 mg/kg) or pterostilbene (doses ranging from 50 to 200 mg/kg) on the percentage of entries into the open arm both in comparison to the saline-treated non-kindled group and to the control PTZ-kindled group (*p* > 0.05). Results are presented in Fig. 4 (one-way ANOVA: percentage of time spent in the open arm, F(5,64) = 2.235, *p* = 0.061; percentage of open arm entries, F(5,64) = 1.806, *p* = 0.124).

### Effect of repeated treatment with pterostilbene on the depressive-like behavior in the PTZ-kindled mice

**Fig. 5:**
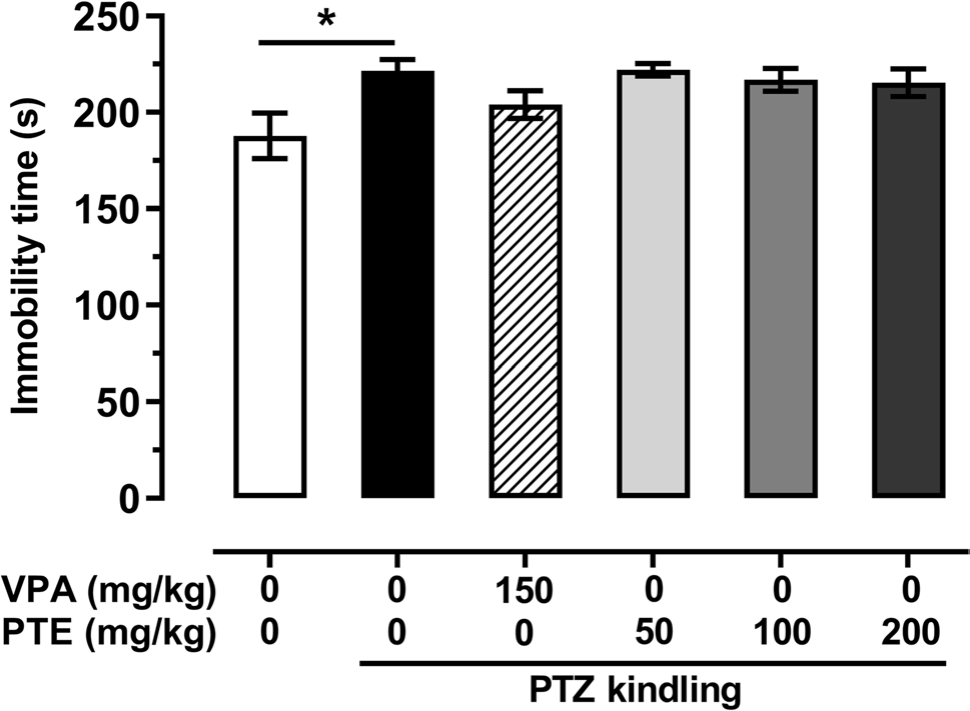
Effect of repeated treatment with pterostilbene on the depressive-like behavior of the PTZ-kindled mice in the forced swim test. PTE, pterostilbene; PTZ, pentetrazol; VPA, valproic acid. Data are presented as means ± SEM, *n* = 8–15 mice per group. Statistical analysis was performed using one-way ANOVA (F(5,64) = 2.898, *p* < 0.021) followed by Dunnett’s multiple comparison test. *p < 0.05

The mean immobility time in the forced swim test in the PTZ-kindled mice was significantly longer than in the saline-treated non-kindled group. In the non-kindled group, it was 187 ± 11.8 s, while in the control group subjected to the PTZ kindling procedure it was increased to 221 ± 5.9 s (*p* < 0.05). Repeated treatment with VPA (150 mg/kg) or pterostilbene at doses ranging from 50 to 200 mg/kg did not significantly affect behavior of the PTZ-kindled mice in the forced swim test. Results of the test are presented in Fig. 5 (one-way ANOVA: F(5,64) = 2.898, *p* < 0.021, with Dunnett’s multiple comparison test).

### Effect of repeated treatment with pterostilbene on GABA and glutamate concentrations in the prefrontal cortex and hippocampus of the PTZ-kindled mice

Neither GABA nor glutamate concentrations in the prefrontal cortex and hippocampus of saline-treated non-kindled mice changed after the forced swim test (*p* > 0.05).

**Fig. 6:**
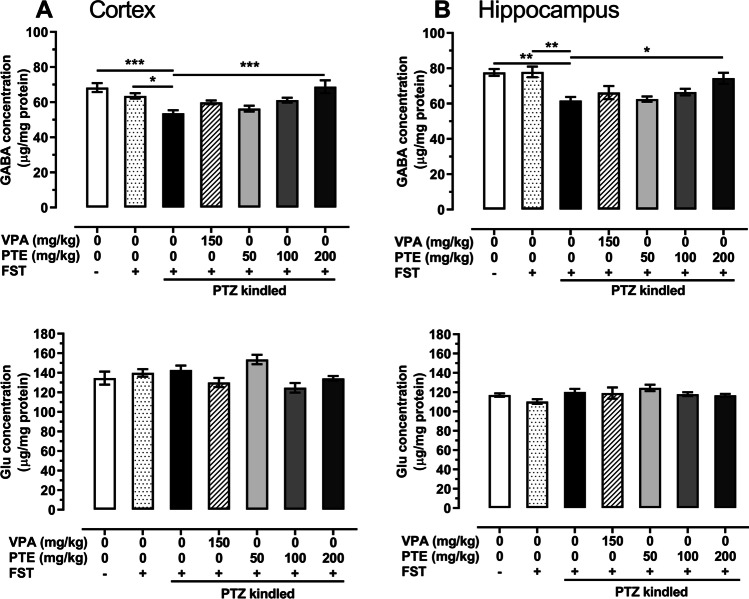
Effect of repeated treatment with pterostilbene on GABA and glutamate concentrations in the prefrontal cortex (panel **A**) and hippocampus (panel **B**) of the PTZ-kindled mice. FST, forced swim test; Glu, glutamate; PTE, pterostilbene; PTZ, pentetrazol; VPA, valproic acid. Data are presented as means ± SEM, *n* = 6 per group. Statistical analysis was performed using one-way ANOVA (cortex: GABA concentration, (F(6,35) = 7.385, *p* < 0.0001, glutamate concentration, F(6,35) = 3.995, *p* = 0.004; hippocampus: GABA concentration, F(6,35) = 7.356, *p* < 0.0001, glutamate concentration, F(6,35) = 1.815, *p* = 0.125) followed by Tukey’s multiple comparison test. *p < 0.05, **p < 0.01, and ***p < 0.001

The PTZ-induced kindling procedure significantly decreased GABA concentration in the prefrontal cortex of mice (vs. both saline-treated non-kindled groups). The mean GABA concentration in the naïve saline-treated group was 68.33 ± 2.5 µg/mg of protein, in the saline-treated group subjected to the forced swim test it was 63.59 ± 1.5 µg/mg of protein, while in the control PTZ-kindled group it was reduced to 53.65 ± 1.8 µg/mg of protein. Repeated treatment with pterostilbene at a dose of 200 mg/kg increased GABA concentration to 68.80 ± 3.6 µg/mg of protein (*p* < 0.001 vs. PTZ-kindled group). There were no statistically significant changes of glutamate level in the prefrontal cortex in the saline-treated PTZ-kindled group (*p* > 0.05 vs. both saline-treated non-kindled groups). Moreover, neither repeated treatment with VPA nor with pterostilbene influenced glutamate level in the prefrontal cortex of the PTZ-kindled mice (*p* > 0.05 vs. PTZ-kindled group). GABA and glutamate concentrations in the prefrontal cortex are presented in Fig. 6A (one-way ANOVA: GABA level, F(6,35) = 7.385, *p* < 0.0001; glutamate level, F(6,35) = 3.995, *p* = 0.004).

Similar changes of neurotransmitter concentrations were also noted in the hippocampus—GABA level in the PTZ-kindled group was significantly lower than in the naïve non-kindled group as well as in the non-kindled group subjected to the forced swim test. Repeated administration of pterostilbene at a dose of 200 mg/kg increased GABA level from 61.70 ± 2.1 µg/mg of protein in the control PTZ-kindled group to 74.35 ± 3.1 µg/mg of protein (*p* < 0.05). The PTZ kindling procedure did not significantly influence glutamate concentration in the hippocampus of mice. Moreover, repeated treatment with VPA or pterostilbene did not change the concentration of this neurotransmitter in the hippocampus of the PTZ-kindled mice (*p* > 0.05 vs. control PTZ-kindled mice). Levels of GABA and glutamate in the hippocampus are presented in Fig. 6B (one-way ANOVA: GABA level, F(6,35) = 7.356, *p* < 0.0001; glutamate level, F(6,35) = 1.815, *p* = 0.125).

### Effect of repeated treatment with pterostilbene on BDNF mRNA expression in the prefrontal cortex and hippocampus of the PTZ-kindled mice

**Fig. 7:**
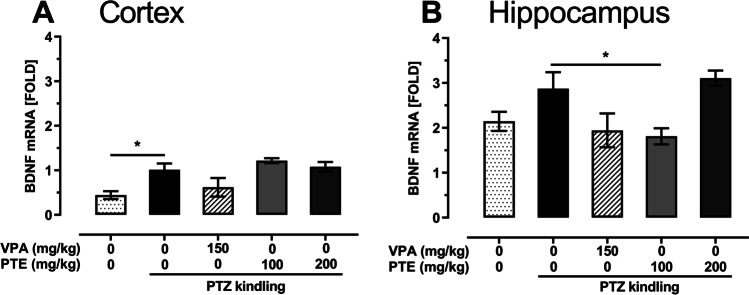
Effect of repeated treatment with pterostilbene on BDNF mRNA expression in the prefrontal cortex (panel **A**) and hippocampus (panel **B**) of the PTZ-kindled mice. PTE, pterostilbene; PTZ, pentetrazol; VPA, valproic acid. Data are presented as means ± SEM, *n* = 3–6 per group. Statistical analysis was performed using one-way ANOVA (cortex: F(4,19) = 5.063, *p* = 0.006; hippocampus: F(4,21) = 4.235, *p* = 0.011) followed by Dunnett’s multiple comparison test. *p < 0.05 vs. saline-treated PTZ-kindled group

One-way ANOVA revealed that the PTZ-induced kindling procedure significantly increased BDNF mRNA expression in the prefrontal cortex (Fig. 7A; one-way ANOVA: F(4,19) = 5.063, *p* = 0.006) but it did not affect the expression in the hippocampus (Fig. 7B; one-way ANOVA: F(4,21) = 4.235, *p* = 0.011). Repeated treatment with VPA (150 mg/kg) or pterostilbene (100 and 200 mg/kg) did not affect BDNF mRNA expression in the prefrontal cortex of the PTZ-kindled mice. In the hippocampus, a statistically significant decrease in BDNF mRNA expression was noted in the group of PTZ-kindled mice repeatedly treated with pterostilbene at a dose of 100 mg/kg (*p* < 0.05 vs. PTZ-kindled group).

### Effect of repeated treatment with pterostilbene on TNF-α, IL-1β, and IL-6 mRNA expression in the prefrontal cortex and hippocampus of the PTZ-kindled mice

**Fig. 8:**
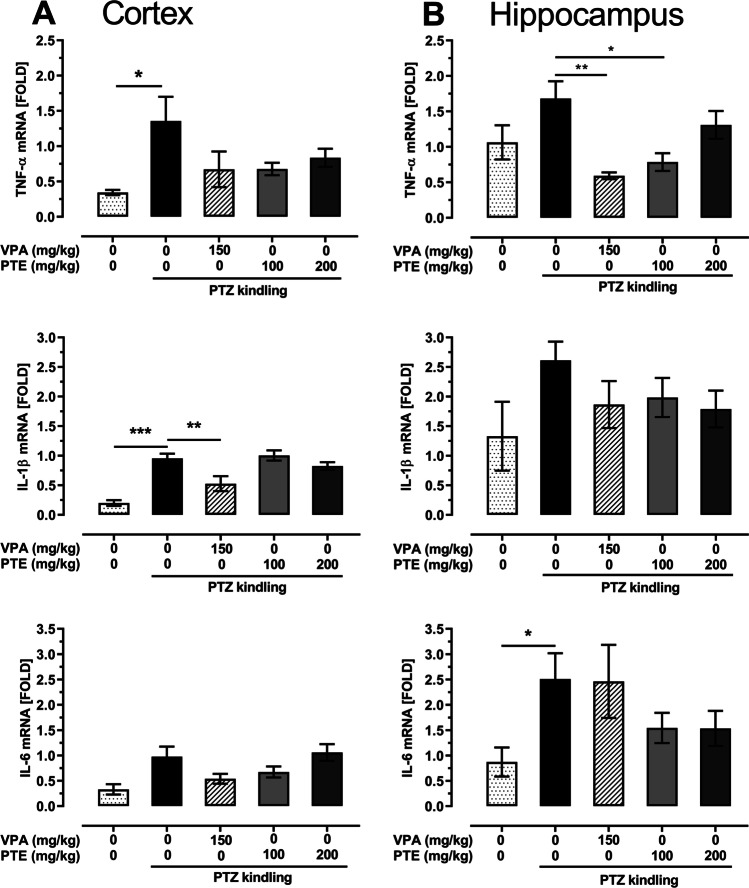
Effect of the repeated treatment with pterostilbene on TNF-α, IL-1β and IL-6 mRNA expression in the prefrontal cortex (panel **A**) and hippocampus (panel **B**) of the PTZ-kindled mice. PTE, pterostilbene; PTZ, pentetrazol; VPA, valproic acid. Data are presented as means ± SEM, *n* = 3–6 per group. Statistical analysis was performed using one-way ANOVA (cortex: TNF-α, F(4,18) = 2.436, *p* = 0.085; IL-1β, F(4,19) = 12.34, *p* < 0.0001; IL-6, F(4,17) = 3.248, *p* = 0.038; hippocampus: TNF-α, F(4, 21) = 4.508, *p* = 0.009; IL-1β, F(4,21) = 1.468, *p* = 0.248; IL-6, F(4,21) = 2.52, *p* = 0.072) followed by Dunnett’s multiple comparison test. *p < 0.05, **p < 0.01, ***p < 0.001 vs. saline-treated PTZ-kindled group

The PTZ-induced kindling procedure caused statistically significant increase in TNF-α (*p* < 0.05 vs. saline-treated non-kindled group) and IL-1β (*p* < 0.001 vs. saline-treated non-kindled group) mRNA expression in the prefrontal cortex of mice. There were not any statistically significant changes of TNF-α and IL-1β mRNA expression caused by repeated treatment with pterostilbene (50–200 mg/kg) in the PTZ-kindled mice, while repeated treatment with VPA (150 mg/kg) significantly reduced IL-1β mRNA expression in the prefrontal cortex of these animals. Statistical analysis did not show any significant changes in IL-6 mRNA expression in the prefrontal cortex. Changes of TNF-α, IL-1β, and IL-6 mRNA expression in the prefrontal cortex are presented in Fig. 8A (one-way ANOVA: TNF-α, F(4,18) = 2.436, *p* = 0.085; IL-1β, F(4,19) = 12.34, *p* < 0.0001; IL-6, F(4,17) = 3.248, *p* = 0.038).

The PTZ-induced kindling procedure did not significantly affect TNF-α and IL-1β mRNA expression (*p* > 0.05 vs. control non-kindled group) but it significantly increased the level of IL-6 mRNA expression (*p* < 0.05 vs. control non-kindled group) in the hippocampus of mice. Statistical analysis revealed significant reduction of TNF-α mRNA expression in the hippocampus of kindled mice repeatedly treated with VPA at a dose of 150 mg/kg (*p* < 0.01 vs. control PTZ-kindled group) as well as with pterostilbene at a dose of 100 mg/kg (*p* < 0.05 vs. control PTZ-kindled group). Neither repeated VPA (150 mg/kg) nor pterostilbene (100–200 mg/kg) treatment significantly affected IL-1β and IL-6 mRNA expression in the hippocampus of the PTZ-kindled mice (*p* > 0.05 vs. control PTZ-kindled mice). TNF-α, IL-1β, and IL-6 mRNA expression in the hippocampus of the PTZ-kindled mice is presented in Fig. 8B (one-way ANOVA: TNF-α, F(4, 21) = 4.508, *p* = 0.009; IL-1β, F(4,21) = 1.468, *p* = 0.248; IL-6, F(4,21) = 2.52, *p* = 0.072).

### Effect of repeated treatment with pterostilbene on *GABRA1A* and *GRIN2B* mRNA expression in the prefrontal cortex and hippocampus of the PTZ-kindled mice

**Fig. 9:**
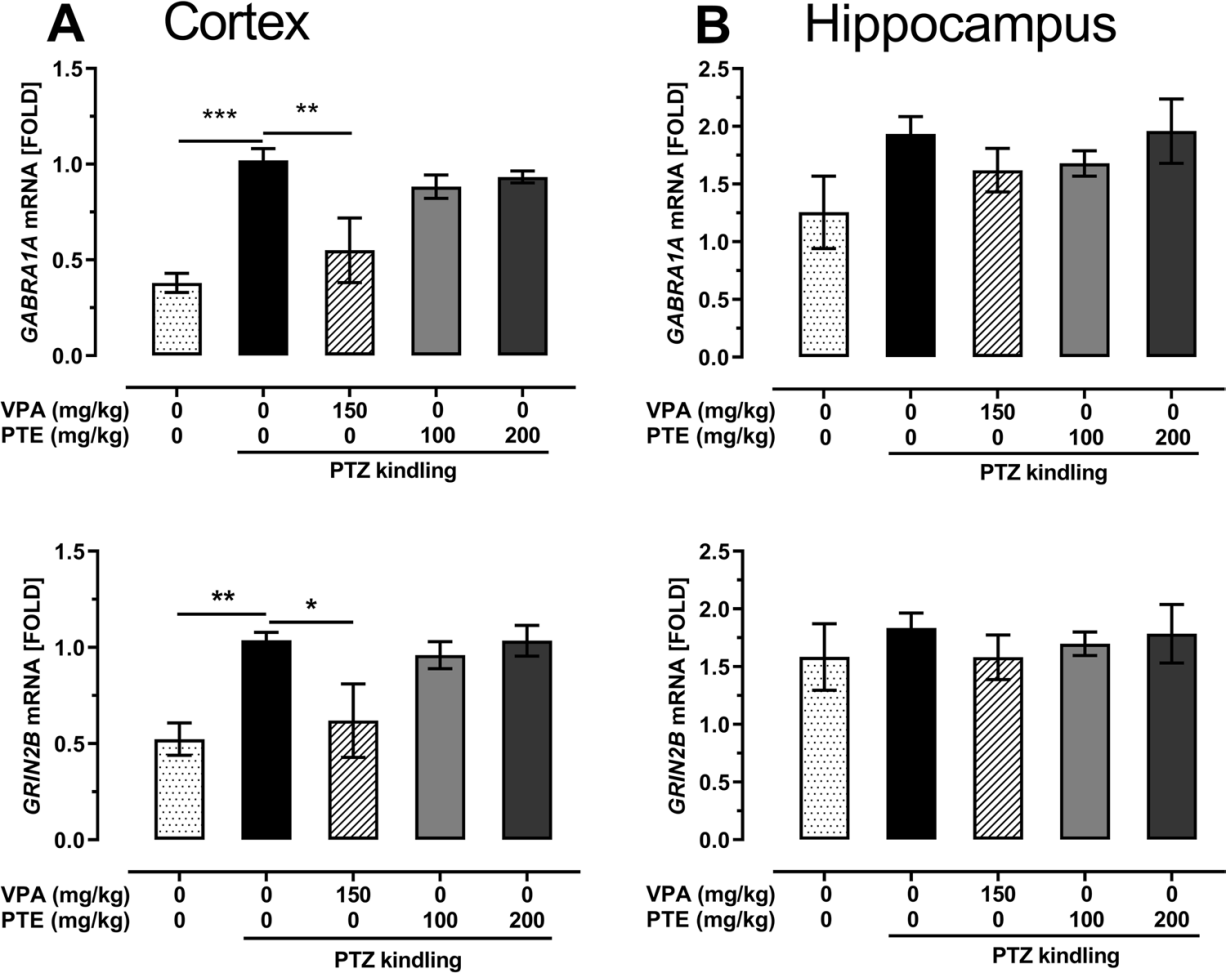
Effect of the repeated treatment with pterostilbene on the *GABRA1A* and *GRIN2B* mRNA expression in the prefrontal cortex (panel **A**) and hippocampus (panel **B**) of the PTZ-kindled mice. PTE, pterostilbene; PTZ, pentetrazol; VPA, valproic acid. Data are presented as means ± SEM, *n* = 3–6 per group. Statistical analysis was performed using one-way ANOVA (cortex: *GABRA1A* mRNA, F(4,19) = 10.03, *p* = 0.0002, *GRIN2B* mRNA, F(4,19) = 5.634, *p* = 0.004; hippocampus: *GABRA1A* mRNA, F(4,22) = 1.613, *p* = 0.207, *GRIN2B* mRNA, F(4,24) = 0.3304, *p* = 0.855) followed by Dunnett’s multiple comparison test. *p < 0.05, **p < 0.01, ***p < 0.001)

Statistical analysis revealed that the PTZ-induced kindling procedure increased the level of *GABRA1A* (*p* < 0.001 vs. control non-kindled group) and *GRIN2B* (*p* < 0.01 vs. control non-kindled group) mRNA expression in the mouse prefrontal cortex (Fig. 9A; one-way ANOVA: *GABRA1A* mRNA, F(4,19) = 10.03, *p* = 0.0002, *GRIN2B* mRNA, F(4,19) = 5.634). Repeated treatment with VPA (150 mg/kg) reduced both *GABRA1A* (*p* < 0.01 vs. PTZ-kindled group) and *GRIN2B* (*p* < 0.05 vs. PTZ-kindled group) mRNA expression in the prefrontal cortex of the PTZ-kindled mice. Pterostilbene administered at doses of 100 and 200 mg/kg affected neither *GABRA1A* nor *GRIN2B* mRNA expression in the brain prefrontal cortex.

We did not note any statistically significant changes of *GABRA1A* and *GRIN2B* mRNA expression in the hippocampus of the PTZ-kindled mice (Fig. 9A; one-way ANOVA: *GABRA1A* mRNA, F(4,22) = 1.613, *p* = 0.207, *GRIN2B* mRNA, F(4,24) = 0.3304, *p* = 0.855). Moreover, their expression was not affected by VPA (150 mg/kg) and pterostilbene (100 and 200 mg/kg).

## Discussion

The present study demonstrated the protective properties of pterostilbene in the PTZ-induced kindling in mice. This model is widely used for the antiepileptic drug screening as well as to evaluate neurobehavioral and neurophysiological consequences of epileptic seizures. Repeated administration of PTZ at a subconvulsive dose leads to the development of seizures that initially manifest as weak myoclonic jerks and then evolve to the generalized clonic-tonic seizure activity (Samokhina and Samokhin 2018). Behavioral changes observed during the kindling procedure arise from some progressive neurophysiological and neurochemical alterations in the brain, including modifications of neurotransmission systems, pro-inflammatory and pro-oxidative changes, neuronal loss, and astrocytosis in the hippocampus (Dhir 2012; Samokhina and Samokhin 2018). Repeated treatment with pterostilbene significantly suppressed the development of epileptic seizures in the PTZ-induced kindling procedure. The present findings demonstrate that pterostilbene acts not only symptomatically suppressing acute seizure activity but also prevents some cellular and molecular changes in the brain of the PTZ-kindled mice (Hoda et al. 2017; Meng et al. 2014; Saha and Chakrabarti 2014). Its activity in these studies results from antioxidant (Saha and Chakrabarti 2014) and anti-inflammatory (Hoda et al. 2017) properties. Moreover, resveratrol reduced the death of neuronal cells in the hippocampus and prevented the blood–brain barrier disruption in the PTZ-kindled animals (Meng et al. 2014).

In our study, behavioral tests for measuring anxiety- and depression-like behaviors in the PTZ-kindled mice were conducted 24 h after the last PTZ injection. Additionally, spontaneous locomotor activity was measured. Although the PTZ-induced kindling procedure did not affect the anxiety-like behavior and spontaneous locomotor activity in mice, it significantly enhanced behavioral despair of animals in the forced swim test, which was evidenced as prolongation of the immobility time. Development of the depressive-like behavior in the PTZ-kindled animals has been also reported in previous studies (Azim et al. 2017; Godlevsky et al. 2014; Kaminski et al. 2020). We did not observe the anxiolytic- and antidepressant-like effects of pterostilbene in the PTZ-kindled mice, although these properties were previously reported in non-epileptic animals (Al Rahim et al. 2013; Yang et al. 2019).

The mechanism of proconvulsant activity of PTZ is not precisely recognized but it is considered to result mainly from the antagonistic activity at the benzodiazepine site in the GABA_A_ receptor complex and attenuation of GABAergic neurotransmission (Samokhina and Samokhin 2018). Therefore, to determine the mechanism of protective activity of pterostilbene, we started from evaluation of its influence on the GABA and glutamate concentrations in the prefrontal cortex and hippocampus of the PTZ-kindled mice. We noted that the PTZ-induced kindling procedure down-regulated GABA level in the prefrontal cortex and hippocampus but did not cause any statistically significant changes of glutamate concentration in these brain structures. Altered levels of neurotransmitters, i.e., a decrease of GABA and an increase of glutamate concentrations, in brains of PTZ-kindled rodents have been reported in numerous studies; however, these parameters were most often evaluated in the whole brain (Koshal and Kumar 2016; Kumar and Kumar 2017; Kumar et al. 2016; Taskıran et al. 2020). If the levels of neurotransmitters were evaluated in the individual brain structures, the changes were variable (Alachkar et al. 2020a; Sejima et al. 1997; Singh et al. 2013). In our study, a decreased GABA concentration in the prefrontal cortex of the saline-treated PTZ-kindled mice confirms the pivotal role of GABAergic neurotransmission in the convulsant action of PTZ, while an increased level of GABA in the prefrontal cortex of the PTZ-kindled mice treated with pterostilbene (200 mg/kg) suggests that this effect might at least partially be responsible for the anticonvulsant effect of the studied compound.

Evaluation of the effect of pterostilbene on the GABAergic and glutamatergic systems in the brain of the PTZ-kindled animals was continued by determination of *GABRA1A* and *GRIN2B* mRNA expression. Since we did not observe any significant differences in GABA and glutamate concentrations between two control non-kindled groups, i.e., naïve saline-treated group vs. saline-treated group subjected to the forced swim test, only one of these groups was selected for further RT-qPCR analysis, i.e., the group that was subjected to the forced swim test. The group treated with pterostilbene at a dose of 50 mg/kg was also eliminated from this analysis because this dose was ineffective—it affected neither the seizure score nor the concentration of neurotransmitters in the brain structures.

*GABRA1A* gene encodes α1 subunit of GABA_A_ receptor. Since the subunit’s construction determines functional and pharmacological properties of the receptors, attenuation of the GABAergic neurotransmission in epileptic individuals might result, among others, from changes in the subunit composition of GABA_A_ receptors. The α1 subunit is considered to be involved in epilepsy/seizures (Chuang and Reddy 2018; Greenfield 2013) and modifications in its expression were noted in different brain structures both in epileptic patients (Kanaumi et al. 2006; Loup et al. 2009) as well as in experimental animals (Brooks-Kayal et al. 1998; Chen et al. 2019; Szyndler et al. 2018). Szyndler et al. (2018) reported an increased α1 subunit expression in the hippocampus (i.e., in the CA1, CA3, and dentate gyrus) of fully PTZ-kindled animals that were sacrificed 48 h after the last PTZ treatment. Acute treatment with PTZ also potentiated the expression of this subunit but the effect was limited to the dentate gyrus only. Changes in the α1 subunit expression in the cortex were not evaluated in that study. On the other hand, Follesa et al. (1999) did not observe any statistically significant changes in amounts of α1 subunit mRNA in the hippocampus and cortex of the PTZ-kindled rats both 3 and 30 days after the last PTZ treatment. In our study, the PTZ kindling procedure significantly up-regulated *GABRA1A* mRNA expression in the prefrontal cortex but did not affect it in the hippocampus. Repeated treatment with VPA reversed the PTZ kindling–induced up-regulation of α1 subunit mRNA expression in the prefrontal cortex. It might be hypothesized that an increase in the *GABRA1A* mRNA expression in the control PTZ-kindled animals is some kind of accommodative modification to prevent seizure activity. An up-regulated α1 subunit mRNA expression might lead to the increased number and density of GABA_A_ receptors in the brain structures and then to the potentiation of GABAergic inhibitory neurotransmission in the brain. Treatment with VPA prevented the development of seizure activity as well as the elevation of *GABRA1A* mRNA expression. The anticonvulsant action of pterostilbene in our study was not associated with changes in *GABRA1A* mRNA expression, although Li et al. (2017) demonstrated an up-regulation of α1 subunit expression in the hippocampus of resveratrol-treated rats with kainic acid-induced epilepsy.

*GRIN2B* gene encodes NR2B subunit of NMDA receptor. Changes in NR2B subunit mRNA and protein expression were noted both in epileptic patients (Mikuni et al. 1999; Zhand et al. 2018) as well as in experimental models of seizures/epilepsy (Bo et al. 2004; Zhu et al. 2015). Western blot analysis revealed increased NR2B protein expression in the hippocampus of the fully PTZ-kindled mice and these changes seem to be long-lasting because they were observed both 6 h and 7 days after the last PTZ administration (Zhu et al. 2015). In our study, RT-qPCR analysis revealed increased *GRIN2B* mRNA expression in the prefrontal cortex of the control PTZ-kindled mice, which confirms that proconvulsant action of PTZ is mediated by modification of not only GABAergic neurotransmission but also the glutamatergic one. VPA treatment abolished the PTZ-induced NR2B subunit mRNA overexpression, which might be a part of the mechanism of its anticonvulsant action. The lack of influence of pterostilbene on *GRIN2B* mRNA expression as well as on the glutamate level in the studied brain structures suggests that its anticonvulsant action is rather not related to the modification of glutamatergic neurotransmission in the brain.

The epileptogenic modifications, including these in the PTZ-induced kindling, are accompanied by increased expression of neurotrophins, i.e., BDNF and/or nerve growth factor (NGF) (Han et al. 2000; Malhi et al. 2014). Numerous studies revealed up-regulation of BDNF level in the brain structures of the PTZ-kindled animals (Han et al. 2000; Malhi et al. 2014), which is in accordance with our results showing a statistically significant up-regulation of BDNF mRNA expression in the prefrontal cortex. A higher BDNF mRNA expression was also visible in the hippocampus of the PTZ-kindled mice but it did not reach statistical significance, which might have been caused by a small number of samples and large variations in the respective experimental groups. Similarly, although VPA treatment decreased BDNF mRNA expression both in the prefrontal cortex and hippocampus of the PTZ-kindled mice, the differences were not statistically significant. The significant reduction in the BDNF mRNA level was noted only in the hippocampus of mice treated with pterostilbene at a dose of 100 mg/kg. However, this change seems to be negligible because similar modifications were observed neither in the hippocampus of mice treated with pterostilbene at the higher dose (i.e., 200 mg/kg) nor in the prefrontal cortex. Moreover, previous studies demonstrated that pterostilbene rather increases BDNF expression (Meng et al. 2019; Yang et al. 2019). A clear determination of BDNF role in the kindling process is difficult because some studies that revealed its protective effect against processes of neuronal damage observed in the experimental models of epilepsy (Biagini et al. 2001; Morimoto et al. 2004).

Previous experimental and clinical studies have provided clear evidence of participation of inflammatory processes in the etiology of epileptic disorders (Chmielewska et al. 2020; Mukhtar 2020) and inflammation is considered a marker of epileptogenesis (Vezzani and Friedman 2011). Moreover, anti-inflammatory compounds have been shown to exert protective effects in the animal models of seizures/epilepsy (Elgarhi et al. 2020; Singh et al. 2019; Vieira et al. 2016). Considering the above conditions, we speculated that the protective effect of pterostilbene in the PTZ kindling in mice might arise from its anti-inflammatory properties which were reported in the previous studies (Choo et al. 2014; Lin et al. 2020; Liu et al. 2019, 2020). In our study, PTZ kindling procedure triggered the inflammatory process in the brain which was manifested by up-regulation of TNF-α, IL-1β, and IL-6 mRNA levels in the prefrontal cortex and/or hippocampus of mice. These findings are consistent with previous results (Gao et al. 2018; Singh et al. 2019; Taskıran et al. 2020). The only statistically significant change of the pro-inflammatory markers in the VPA- and pterostilbene-treated PTZ-kindled mice was the reduction of TNF-α mRNA expression in the hippocampus. Although the levels of mRNA expression of cytokines in the studied brain structures were in most cases lower than in the control PTZ-kindled group, these differences did not reach statistical significance. As in the case of BDNF, it might have been caused by a small number of samples and large variations. Previous studies revealed that anti-inflammatory effect of pterostilbene might be mediated by inhibition of nitric oxide production (Hou et al. 2015). Moreover, nitric oxide signaling affects various neurotransmission systems in the brain (Ferraro and Sardo 2004; Kano et al. 1998; Kiss and Vizi 2001) and therefore might affect seizure activity (Zamanian et al. 2020). Anti-inflammatory mechanism of the anticonvulsant action of pterostilbene in the PTZ kindling model cannot be excluded. Its influence on pro-inflammatory cytokines as well as on BDNF expression in epileptic disorders should be further evaluated.

In conclusion, our study revealed the protective activity of pterostilbene in the PTZ-induced kindling in mice. Our previous study showed also its anticonvulsant effect in the acute seizure tests (Nieoczym et al. 2019a). These results indicate that pterostilbene is worthy of attention in the search for new compounds with anticonvulsant and antiepileptogenic potential. Our study has not fully explained the mechanisms of the protective action of pterostilbene in the PTZ kindling. The increase in GABA concentration as well as the downregulation of BDNF and TNF-α expression in brain structures possibly contributed to this beneficial effect. Further studies on properties of pterostilbene in other experimental models of seizures and epilepsy are needed.
